# Middle Eastern Plant Extracts: An Alternative to Modern Medicine Problems

**DOI:** 10.3390/molecules25051126

**Published:** 2020-03-03

**Authors:** Disha Varijakzhan, Chou-Min Chong, Aisha Abushelaibi, Kok-Song Lai, Swee-Hua Erin Lim

**Affiliations:** 1 Institute of Bioscience, Universiti Putra Malaysia, Serdang 43400, Selangor, Malaysia; dishavarijakzhan@gmail.com (D.V.); choumin@upm.edu.my (C.-M.C.); 2 Department of Aquaculture, Faculty of Agriculture, Universiti Putra Malaysia, Serdang 43400, Selangor, Malaysia; 3 Health Sciences Division, Abu Dhabi Women’s College, Higher Colleges of Technology, Abu Dhabi 41012, UAE; aabushelaibi@hct.ac.ae (A.A.); lkoksong@hct.ac.ae (K.-S.L.)

**Keywords:** Middle East, plant extracts, bioactivities, antimicrobial activity, anti-cancer

## Abstract

Middle Eastern countries are primarily known for their dry sand deserts; however, they have a wider physiographic range which includes upland plateau and mountain ranges. The Middle East is home to various types of plants, such as *Phoenix dactylifera* (date palm tree), *Scrophularia striata* (herbaceous plants), and *Opuntia ficus-indica* (cactus). These plants have been found to have various types of bioactivities, such as antimicrobial activities against both bacteria and fungi, in addition to exhibiting anti-inflammatory effects and anti-cancer characteristics which can be utilized in the clinical setting for treatment. Due to limited reviews focusing on plant extracts from the Middle East, we aim to provide a discourse on plants from this region which have various bioactivities and to provide information on the compounds that can be identified from these plants. This is to enhance our understanding to improve modern medicine problems such as antimicrobial resistance and to find an alternative cure for cancer. It is hoped that the collation of information from this review will enable an assessment of the direct role of Middle Eastern plants in providing therapeutic options to address the predicaments in the medical field.

## 1. Introduction

**Figure 1:**
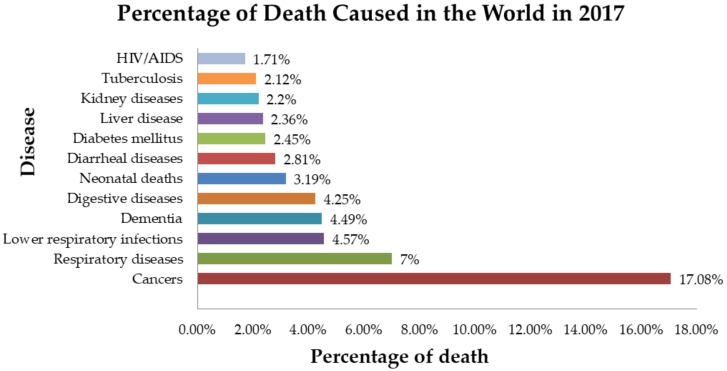
Percentage of deaths in the world in 2017 caused by communicable and non-communicable diseases [8].

After the Industrial Revolution in the 18th century, modern medicine as we know it began to emerge. There were a few achievements that led to the development of the field of modern medicine, which would be the discovery of the small pox vaccination by Edward Jenner in 1796, followed by inventions such as the stethoscope and syringes in 1816 and 1853 by Rene Laennec, and Charles Gabriel Pravaz together with Alexander Wood, respectively [1]. During the 19th century and onwards, there was a major breakthrough in identifying the causes of illness and preventing illnesses, whereby various human infectious disease-causing agents were identified [2]. This was made possible when Louis Pasteur in 1857 proved that germs are the causative agent of diseases, followed by Robert Koch, who postulated the germ theory of disease; this is known as Koch’s postulate, which was demonstrated using *Mycobacterium tuberculosis* [3]. Following that, the discovery of penicillin, an antibiotic, by Alexander Fleming in 1928 was another major contribution that has led to the revolution of the medical field [1]. These discoveries are foundation of modern medicine and led to the discovery of various illnesses, their causative agents, and also the discovery and manufacture of antimicrobials, which have led to a reduction in mortality. Regardless of continuous and progressive achievements in the area of medical field, there are impending threats which cannot be overlooked. One such threat would be the rise in antimicrobial resistance among pathogens due to over-prescription and also the development of resistance among cancer-curing drugs through DNA mutation by the cancerous cells [4,5,6,7]. Figure 1, below, shows the percentage of deaths caused in the world due to communicable and non-communicable diseases in 2017 [8].

According to a report published by World Health Organization (WHO), by 2050, there will be approximately 10 million deaths caused by the drug-resistant pathogens and common diseases such as respiratory tract infections and urinary tract infections every year, which will be higher than the number of deaths caused by cancer [9,10]. Hence, it is necessary to look into alternative solutions, such as natural products, for example, plant extracts, to overcome impending threats in the medical field [11,12]. It has been widely reported that plant extracts such as essential oils contain various bioactive compounds which are responsible for bioactivities, such as antimicrobial, anticancer, and anti-inflammatory agents [6,12,13,14,15]. As nature is rich with different plants, researchers have focused their attention towards plant extracts as an alternative to synthetic drugs to be applied in the clinical setting [16]. An example of these plants would be those plants grown in the Middle Eastern region. They are known to be able to tolerate extreme weather conditions. It is known that the plant extracts synthesized by plants are affected by environmental factors, such as the temperature and the availability of water [17,18]. Therefore, the extracts from plants obtained from the Middle Eastern region may provide an array of different compounds with greater variety of bioactivities due to the environmental conditions surrounding these plants.

The Middle East is known as the driest region in the word and consists of Bahrain, Cyprus, Egypt, Iran, Iraq, Israel, Jordan, Kuwait, Lebanon, Oman, Palestine, Qatar, Saudi Arabia, Syria, Turkey, United Arab Emirates, and Yemen [19,20]. However, in fact, the Middle East has diverse physiography, ranging from large gravel and sandy deserts to upland plateau and mountain ranges [21]. Apart from geography, the climate conditions also vary depending on the season, where during summer, the temperature usually will be in the range of 38–42 °C, whereas during winter the temperature may drop to 14 °C [21]. Despite being known as the driest region in the world, there are approximately 13,500 species of plants that can be found in Middle East countries. Plants from genera *Acantholimon, Acanthophyllum, Astragalus, Centaurea, Cousinia, Dionysia, Nepeta, Phlomis, Salvia, Saponaria, Silene, Stachys, Thymus,* and *Verbascum* are example of plants that are predominantly found in these regions [21]. Iraq consists of rich and diverse species of plants, with approximately 3300 species of them found in deserts, mountains, and plain range.

Plants from this region are less explored, but their counterpart plants from other regions have or are being widely explored for their application in plant extracts. One example would be the date palm tree from the African region, which has been tested for its antibacterial activity and has been reported to have activity against Enterobacteriaceae, such as pathogenic strains of *Escherichia coli*, *Klebsiella pneumoniae*, *Salmonella typhi*, and *Proteus mirabilis*, but, other than *E. coli* and *K. pneumoniae* strains, date palm trees from the Middle East have yet to be tested for antibacterial activity against other Enterobacteriaceae [22]. The native plants of the Middle East have also been used as a form of local traditional medicine by the natives, indicating the ability of the Middle Eastern plants to heal various illnesses. Hence, in this review, we will be focusing on Middle Eastern plant extracts as a potential solution for the challenges faced in the clinical setting, such as the increase in antimicrobial resistance. We will also provide information on various bioactivities, such as anti-cancer, antimalarial, and anti-inflammatory activities, along with the compounds responsible for these bioactivities from the plants.

## 2. Traditional Herbal Medicines

Traditional medicine is a term used to refer to any non-Western medical practice and the practice of traditional medicines dates back to prehistoric times, whereby fossil records indicate that humans have used plants as a medicinal source since 60,000 years ago [23,24,25]. Traditional Chinese medicine, Ayurveda, traditional Korean medicine, Arabic unani medicine, and Iranian traditional medicine are a few examples of traditional medicines which have been practiced all over the world [26]. They consist of medicines which are made from leaves, roots, herbs, barks, and minerals, which can be found abundantly in nature and they work together synergistically [27]. The application of traditional medicine as a remedy, known as “alternative” or “complementary”, medicine has been widely accepted in both developed and developing countries [27].

As per the reports, approximately four billion people from developing countries (approximately 80% of the world population) rely on traditional medicine as a main source of cure for ailments. Meanwhile, in countries such as the United States of America, Canada, and France, it is estimated that 42%, 70%, and 75% of their populations, respectively, have used herbal medicines at least once [27,28,29,30]. The shift to traditional medicines from conventional modern medicine was observed due to the natural ways of treating various diseases that can improve the healthcare of the general public and also due to the presence of legislation and regulations which are responsible for ensuring the quality and safety of traditional medicines in some countries, such as in Europe and the United Kingdom, thus promoting usage among the common public [27,31].

Traditional medicines have been used as a reference to isolate medicinal value compounds from plants by researchers to be applied in modern medicine. Such a discovery would be the discovery of morphine from the tarry poppy seed juice, by Friedrich Sertürner, from the plant opium poppy [32,33,34,35]. Other than that, there are numerous bioactive compounds, especially alkaloids such as caffeine and nicotine, that have been extracted from natural products [36,37]. Besides that, anti-cancer, anti-hypertensive, and anti-migraine medications have also been extracted from plants. For instance, the Pacific yew tree found in the northwest region of the United States of America was used by the natives as a healing medicine. The tree was discovered to have a taxol compound that has significant anti-cancer activity; this has been proven to be a promising cure for breast and ovarian cancer [38].

Ayurveda and Chinese traditional medicine are well established and widely practiced forms of traditional medicines with a lot of research and publications. However, there are some practiced traditional medicines which have been less reviewed compared to Ayurveda and Chinese traditional medicines, but with equally important information. One such example would be the Middle Eastern-based plant extracts, which are rich in different types of compounds that can be grouped based on their chemical structures and have high bioactive activities.

## 3. Phytochemistry of Plant Extracts

The extraction of plants can be conducted in a few methodologies. Hydro distillation is one of the methods used to isolate the plant extract [39,40]. The plant material will be heated in water, followed by the liquefaction of the vapors. Solvent extraction is another classic method which is commonly applied to obtain plant extracts. Solvents such as acetone, ethanol, methanol, and hexane are used [41,42]. This technique is applied when the part of the plant which is to be extracted is fragile and easily disrupted, or where heat or steam techniques cannot be applied [43]. It is important to consider the polarity of the compound—that is, considering the ability of the solute to dissolve in a provided solvent [44]. Polar solvents tend to dissolve ionic or highly polar solutes, whereas non-polar solvents tend to dissolve non-polar solutes. Hence, the extraction from the plants will be done from highly polar solvent, followed by low polar solvent, and finally non-polar solvent. The botanical sample is combined with a solvent and then mildly heated, followed by the filtration and evaporation of the solvents using a rotary evaporator. Following the extraction of the plants, the compound of the extracts must be identified. There are a few techniques where the isolation and identification of the compounds can be conducted. The techniques can be grouped as chromatography, electrophoresis, and spectroscopic techniques. Table 1 below shows the summary of the techniques.

## 4. Bioactivity of Plant Extracts from Middle East

The Middle East is home to different species of plants, which exist primarily in that region despite having an extreme climate. Table 2 below lists plants from this region and the bioactivities of the compounds obtained from the plant extracts, which can be utilized to overcome medical predicaments such as resistance to antibacterial drugs, cure for cancer, and antifungal, to name a few.

### 4.1. Antimicrobial Activity

There are various plant extracts that have indicated antimicrobial activity against a wide range of microorganisms, such as bacteria, fungi, and viruses [6]. The antimicrobial activity of these extracts is attributed to the presence of various groups of compounds, such as phenolics, alkaloids, saponins, terpenes, lipids, and carbohydrates [76,77]. The compounds present in the plant extracts are dependent on the types of solvents used during the extraction process [78]. Compounds which belong to the phenol and terpene groups are known widely to have antimicrobial activity [6]. These phenolic compounds act on the bacteria by altering the permeability of the plasma membrane of bacterial cells, altering enzymes in the cells or through modifying the rigidity of the cell wall; this results in the loss of integrity in the cell membrane [79]. These are known to be irreversible cell damages. We will be discussing various plants which are found in the Middle Eastern region that have antimicrobial activity against various pathogens.

Dates (*Phoenix dactylifera*) have been identified to exhibit antimicrobial activity from various parts of the tree, such as the fruit and seeds, due to the presence of phenolic compounds [80,81]. Date seeds from Saudi Arabia have been tested against *Klebsiella pneumoniae* and *Escherichia coli* to determine their antimicrobial activity and the zone of inhibition obtained was compared with known antibiotics [51]. From the study, it was found that the zone of inhibition of date seed water extract was comparable to cefotaxime (16.33 mm) on *K. pneumoniae* (16.351 mm), whereas for *E. coli* (10.00 mm) the zone of inhibition was similar to amikacin (14.00 mm) and aztreonam (15.33 mm). The antimicrobial activities of date seeds are due to the presence of phenolic compounds, such as p-coumaric, ferulic and sinapic acids, flavonoids, and procyanidins [50]. Three types of dates from Oman (Mabseeli, Um-sellah, and Shahal) have also been reported to have high phenolic content, rich in antioxidant and strong antimicrobial activity [82].

The leaves of the date palm are rich in phenolic compounds, whereby the highest phenolic content can be obtained when extracted using methanol [81]. The leaves of date palm have been shown to exhibit antimicrobial activity against both Gram-positive and Gram-negative bacteria. A study was conducted to test the antimicrobial activity of three different cultivars of date palm leaves, Sukkaria, Hillaliah, and Hoshana, against Gram-positive bacterial strains (*Staphylococcus aureus* ATCC 25923, *Staphylococcus epidermidis* ATCC 12228, *Enterococcus faecalis* ATCC 29212, and *Bacillus cereus* ATCC 10876) and Gram-negative bacterial strains (*Klebsiella pneumoniae* ATCC 700603 and *Escherichia coli* ATCC 35218) [83]. All three methanol leaf extracts exhibited a varied degree of antibacterial properties. The Gram-positive bacteria were more susceptible towards the methanol leaves extracts compared to Gram-negative bacteria. *S. aureus* was highly susceptible to the leaves extract compared to the other Gram-positive strains, whereas *E. faecalis* was the least sensitive. *K. pneumoniae* exhibited no susceptibility against all three extracts, however, *E. coli* showed a small amount of susceptibility towards Hillaliah leaf methanol extracts. The methanol extracts were further examined for antioxidant activity and Hillaliah methanol extract showed the highest antioxidant activity (93.2%) at a concentration of 100 mg/mL, and Hoshana and Sukkaria recorded 88.9% at a concentration of 100 mg/mL. Terpenoids, phenolic, and flavonoids, which have antimicrobial activity, also contribute to the antioxidant activity which was identified through 2,2-diphenyl-1-picryl-hydrazyl-hydrate (DPPH) radical scavenging activity, reducing power assay, total antioxidant capacity, and reduction of ferric ions assays [84].

Other than the date palm, the phytochemical analysis of figwort (*Scrophularia* sp.) plant extract from various parts, such as the stem, rhizome, and seed, showed that the extracts contain phenolic compounds, which is attributed to the antimicrobial activity [54,55]. The phenolic content of the extracts was found to be higher in the polar extracts such as methanol compared to the non-polar extracts such as water [54]. The figwort plant is commonly found in Iran, distributed around mountain range and deserts [55]. A study was conducted to identify the antimicrobial activity of figworts (*S. striata*) against oral pathogens, *Actinomyces viscous* PTCC (Persian Type Culture Collection) 1202, *Streptococcus mutans* PTCC 1683, *Streptococcus sobrinus* PTCC 1601, *Lactobacillus fermentum* PTCC 1638, *Lactobacillus casei* subsp *casei* PTCC 1608, and *Eikenella corrodens* PTCC 1391 [85]. The extracts of the plants were obtained from hydro-alcoholic (ethanol:water: 50:50) extraction. The inhibition zone of *S. striata* at 100% *w*/*v* was less significant when compared to the control (Chlorhexidine). The zone of inhibition of *S. striata* extract was higher than Irsha mouthwash and tooth brush tree for both *L. casei* and *L. fermentum*, whereas the zone of inhibition of *S. sobrinus* and *A. viscous* was equal to Irsha but less than the tooth brush tree. For *S. mutans* the zone of inhibition was less than the tooth brush tree but more than Irsha, and finally against *E. corrodens* the zone of inhibition was equal to the tooth brush tree but more than Irsha. The antibacterial activity of *S. striata* might be due to the presence of phenolic acids, such as phenylethanoids, and phenylpropanoids, such as flavonoids. Phenylethanoids are derived from benzoic acid and are commonly found in *Scrophularia* sp., whereas phenylpropanoids such as flavonoids are commonly found in plant extracts [86,87,88]. As these compounds belong to the phenolic group, they react with the bacteria by altering the membrane permeability, as mentioned above. 

Water figwort (*Scrophularia umbrosa Boiss*) is a plant native to Iran, and has been reported to have anti-malarial activity [55]. The rhizome from water figwort was treated using dichloromethane, n-hexane, and methanol in one study. The dichloromethane rhizome extract showcased moderate anti-malarial activity, whereas both n-hexane and methanol extracts did not exhibit any anti-malarial activity [55]. The assessment of the anti-malarial activity was conducted using in vitro β-hematin formation assay. At concentrations greater than 3 mg/mL, dichloromethane rhizome extract exhibited potent anti-malarial activity by inhibiting the formation of heme bio crystallization. The gas chromatography-mass spectrometry (GC-MS) analysis was conducted on the extract of dichloromethane and identified linoleic acid (44.61%) and cinnamic acid (27.07%), which resulted in the antimalarial activity. Cinnamic acid is a derivative of phenyl belonging to the group of phenylpropanoid, whereas linoleic acid belongs to the unsaturated fatty acid group [86,89,90]. The mode of action of linoleic acid against bacterial pathogens is by inhibiting the FabI enzyme, which is responsible for the synthesis of bacterial fatty acids [91]. However, the mechanism of anti-malarial activity specifically by these compounds has yet to be determined.

Meswak (*Salvadora persica* L.) is a plant which is commonly related to oral hygiene and is known to contain compounds such as silica, resin, alkaloids, and vitamin C [92]. Meswak grows mainly in Saudi Arabia but also can be found in other regions of the Middle East. Roots and stems of Meswak plants contain silica and resin, where they can form a protective coat over an enamel of the teeth, thus protecting the teeth from microbial activities which might lead to the formation of caries and gingivitis [63,64,65,93]. The silica acts as an abrasive material, where it deposits on the surface of the teeth, whereas resins forms a layer over the enamel, acting as a physical barrier [92].The roots and twigs of Meswak were soaked in sterile distilled water, 96% ethanol, ethyl acetate, and 2% acetic acid to obtain extracts [94]. These extracts then were tested against oral microorganisms *Actinobacillus actinomycetemcomitans* ATCC 43717, *Actinomyces naeslundii*, *Candida albicans* ATCC 90028, *Lactobacillus acidophilus* CCUG 5917, *Porphyromonas gingivalis* W50 Black, *Prevotella intermedia* VPI 4197, and *Streptococcus mutans* CCUG 11877. From the study conducted, *S. mutans* were the most sensitive strain to all the extracts, however, *L. acidophilus* was sensitive only to the root-ethanolic extract. Compared with all the solvent extracts, ethanolic extracts exhibited the strongest antimicrobial activity. Both twigs and roots ethanol extracts were able to inhibit the growth of *C. albicans*, indicating its capability as an anti-fungal agent. Compounds such as *N*-benzylbenzamide, decane, and stigmasterol have been revealed to possess antimicrobial properties, and they are widely found in the stem region of the Meswak plant [63,93]. The compounds that have been identified belong to several groups, where the decane is a hydrocarbon, stigmasterol is a plant sterol, and *N-*benzylenzamide is an amide, and all these compounds have been identified to contain antimicrobial activity [92]. The mechanism of anti-fungal activity from a plant extract is via inhibition of the synthesis of the cell wall and growth of the hyphae of fungus [95]. However, the mechanisms of these specific compounds against bacteria and fungus are yet to be determined. The plant extract of Meswak also has anti-viral properties. The anti-viral activity is due to the presence of the compound benzylisothiocyanate, an isothiocyanate [65]. The plant extract acts on the virus by inactivating the virus particles; this is done by interfering with the adsorption step of the virus to a cell [96]. The Meswak plant stick obtained from Saudi Arabia has exhibited anti-viral property against Herpes simplex virus [97]. The ethanol extract of the plant was tested on baby hamster kidney cells which were infected with Herpes simpex virus. From the study, it was found that all the ethanol extracts at different concentration of Meswak were able to inhibit the growth of herpes simplex virus. The cytolytic activity of the virus reduced in the presence of 0.5%, 1.0%, and 1.5% of the extract concentration post 10 min treatment. However, at 5.0% of Meswak extract, the cytolytic activity was reduced tremendously. Thus, it can be deduced that the ethanol extract of Meswak is able to inhibit the replication of the virus. The compounds which are responsible for the anti-viral activity would be benzyl nitrate and benzylisothiocyanate [97]. Hence, the Meswak plant is also known to be effective anti-viral agent, apart from antibacterial agent.

Savory of crete (*Satureja thymbra*) is an herbaceous plant grown mainly in the Middle East region and has been reported to have anti-viral activity. The plant has been used traditionally to treat stomach and intestinal disorders, such as cramps and indigestion [74]. The plant consists of compounds such as thymol, carvacrol, *p*-cymene, γ-terpinene, borneol, caryophyllene, and bicyclogermacrene as major compounds [74]. Thymol and carvacrol are monoterpenoid phenol compounds, whereas *p*-cymene, γ-terpinene, borneol, caryophyllene, and bicyclogermacrene are terpene compounds. Ethanol extract of savory of crete leaves has been used against monkey kidney cell line infected with Herpes simplex virus type 1 [98]. From the study, it was found that ethanol extract at a concentration of 0.25 mg/mL is able to inhibit 50% of the viral growth, indicating the ability of the extract to be utilized as an anti-viral agent. The result obtained was confirmed by another researcher, who identified that 50% of the viral growth of Herpes simplex virus type-1 was inhibited at a concentration of 0.22 mg/mL [75]. Phenolic compounds found in the extract act on the viral particles by interacting with the glycoprotein of the viral envelope or by inhibiting the polymerase of the viral particles, thus interfering with the synthesis of the viral genome, whereas the terpenes inhibit the virus by interfering with the envelope structure, which is required for the adsorption of the cells to the host cells to cause infection [99,100]. As the ethanol extract of the leaves of Savory of crete consists of different types of compounds, it has been shown to be an effective anti-viral due to its ability to inactivate the viral particles using different mechanisms.

The anti-fungal property of a plant extract is determined by the presence of compounds such as phenols, tannins, flavonoids, terpenoids, and saponins, and the methanol extract of plants has been recorded to contain high amount of these compounds [101,102]. Chicory (*Cichorium intybus*) is a plant that originated from the Iranian region and has been reported to have anti-fungal property [103]. The leaves of the chicory plant soaked in ethanol were tested against the candidiasis pathogen, *Candida glabrata* and *Candida krusei* [69]. From the study, it was found that *C. krusei* is more sensitive to the chicory ethanol extract compared to *C. glabrata*, as *C. krusei* growth was inhibited at a low concentration of 50 µg/mL, whereas growth of *C. glabrata* was inhibited at a concentration of 100 µg/mL. The anti-fungal activity of chicory leaves extract is due to the presence of compounds such as lactucin, lactucopicrin, deoxylactucin, and β-1,3-dihydrolactucin [69,104]. A similar study was conducted on both the *Candida* sp., and similar results were recorded [68]. All the compounds identified from the extract are sesquiterpenes; these compounds inhibit the growth of yeast by altering the cell structure of the *Candida* cells, resulting in the leakage of intracellular cellular content of the cells [101,105].

### 4.2. Anti-Cancer and Anti-Tumor Activity

Other than antimicrobial activities such as antibacterial, antifungal, and antimalarial bioactivities, plant extracts from the Middle East are known to have anti-cancer and anti-tumor properties, with the added ability to also enhance the immune system and induce apoptosis among carcinoma cells; this will enable the possibility of curing cancers and tumors, without much dependence on chemotherapy agents [49]. The anti-cancer and anti-tumor activities in the plant extract are due to the presence of various groups of compounds, such as alkaloids, diterpenes, diterpenoquinone, purine-based compounds, lactonic sesquiterpene, proteins, and macrolides [106]. These compounds are able to inhibit the mitotic cycle of cells or the formation of reversible DNA strand breakage in normal cell cycle, inhibit polymerization of tubulin or by apoptotic death of the cell [106].

Ajwa, a variety of palm date that can be obtained from Saudi Arabia, is known to exhibit anti-cancer properties when tested against cancer cell lines due to its high polyphenolic content [48]. The ethanol extract of the pulp of Ajwa dates was tested against human hepatocellular carcinoma (HCC) HepG2 cells to identify the apoptosis-inducing effect [53]. From the study, it was found that compound β-D-glucan is an active compound found in the ethanol Ajwa pulp extract. It was found that the ethanol Ajwa pulp extract had the ability to induce apoptosis of HepG2 cells in a time- and dose-dependent manner. The HepG2 cell line is human liver carcinoma cells. The extract was able to impair the proliferation of hepatocellular carcinoma (HCC) cell growth, whereby a great effect was observed when the cell line was treated for 48 h at a concentration of 10 mg/mL, 15 mg/mL, 20 mg/mL, 25 mg/mL, and 30 mg/mL. Moreover, the extract showed only low toxicity against Vero cell line, where the survival of the Vero cell line was 98.4%, 96.6%, and 94.8% at 10 mg/mL, 15 mg/mL, and 20 mg/mL, respectively. The extract also exhibited DNA fragmentation in the treated cells. The Ajwa pulp extract was also able to induce intracellular generation of reactive oxygen species (ROS) within the HCC-treated cells and the extract was seen to modulate the expression of tumor suppressor genes such as CHEK2, ATM, and TP53 from the TP53 pathway. Furthermore, the Ajwa date pulp ethanol extract has been shown to effectively exhibit anti-cancer activity by a few mechanisms; that is, fragmentation of DNA, and generation of ROS such as superoxides and hydrogen peroxides, which will result in damage to lipids, proteins, and DNA of a cell and suppression of genes responsible for the growth of tumor cells [53,107].

Other than antimicrobial activity, *S. striata* is also known to have anti-cancer properties against Human Astrocytoma Cell Line (1321), which is from the brain. In a study conducted by A. Lajimi and colleagues (2010) [57], aerial parts of the plants, which are the seeds and the leaves, were studied for anti-cancer properties on Human Astrocytoma Cell Line (1321). The extracts were water extracts, which were divided into filtered extract and unfiltered extract. At LC50 (5.5 µg/mL), filtered leaf extract was able to inhibit the proliferation of cell line 1321, whereas the seed extracts had no cytotoxic effect. The unfiltered leaf extract exhibited both inhibitory effects and stimulatory effects on the proliferation of the cell line 1321. However, the stimulatory effect on the proliferation of the cell line reduced with an increase in incubation period. Nevertheless, the stimulatory effect of the proliferation of cell line was not observed in the filtered extract, indicating that the compound responsible for the stimulatory effect was eliminated through the filtration process. From the flow cytometry experiment, it was found that the 1321 cell line treated with filtered leaf extract induced apoptosis, indicating that apoptosis is the mechanism by which the extract caused death of the cancer cells. The anti-cancer property from *S. striata* can be due to the presence of monoterpenoid compounds such as iridoid glycosides, especially aucubin and catalpol [108]. The compound iridoid glycosides have been known to have anti-cancer properties against various cancer cells, such as breast cancer, renal cancer, and lung cancer [107].

The plant *Scrophularia* has been identified for various bioactivities, such as antimicrobial activity and also anti-cancer and anti-tumor. A similar study was conducted against human breast cancer cell line using methanolic sub-fractions (Fa, Fb, Fc, and Fd) of *Scrophularia oxysepala* [109]. The amount of the sub-fractions was: 1) Fa: 68 mg; 2) Fb: 85 mg; 3) Fc: 60 mg; and 4) Fd: 53 mg. The effect of the sub-fractions was tested on human breast cancer cell line (MCF-7) and mouse fibroscarcoma cell line (WEHI-164) and all the sub-fractions resulted in a dose-dependent reduction in cell viability, and compared among the four sub-fractions, Fa and Fb exhibited the highest cytotoxicity on the cells. The sub-fractions were then tested to identify the cause of death of the cells and identified that all four sub-fractions caused apoptosis at a higher rate. The sub-fractions were also tested with the normal mouse control cell line L929 and less apoptosis was induced by the extracts compared to MCF-7 and WEHI-164.

Cactus (*Opuntia ficus-indica*) is another common plant found in the dry and desert area, belonging to the family Cactaceae, and has 130 genera with about 1500 species that have been identified to have various bioactivities, including anti-cancer properties [110]. The methanol extract of cactus roots has been used to study the effect on ethanol-induced ulcers in rats [61]. The methanolic root extract of *O. ficus-indica* has a high total phenolic content and results in high anti-oxidant activity by inactivating genotoxic molecules such as reactive oxygen species [111]. Rats treated with 80% ethanol showed an increase in the size of the gastric lesion in the stomach, and when the rats were treated with the methanol extract of the root at concentrations of 200 mg/kg, 400 mg/kg, and 800 mg/kg, showed a reduction in the ulcer lesion by 49.21%, 83.13%, and 92.59%, respectively. There was also an increase in mucus production observed in the stomach of the rats treated with 800 mg/kg of the extract. Therefore, the extract can be utilized as an anti-ulcer remedy and has a gastro-protective effect on ethanol-induced gastric lesions in rats.

Meswak plant extract has been identified to have antitumor properties due to the presence of high phenolic and flavonoid contents, along with antimicrobial activity [67]. In a study, the stems of Meswak plant were dried and extracted in 90% ethanol. The ethanol extract of stems of Meswak was found to contain sterols, tannins, coumarins, alkaloids, and glycosides, and these compounds contain antitumor properties [112,113]. The extract was tested on murine mouse melanoma and the extract was able to delay the growth of tumors. This was assessed by determining the time taken for the tumor to reach double the size of the initial treatment (VDT) and the difference in time (days) for the treated and untreated tumors to reach five-times the treatment volume. From the study, it was found that the extracts managed to increase the VDT and growth delay (GD), thus delaying the growth of antitumor. The mechanism of action of these compounds against tumor cells would be inhibiting the topoisomerase enzymes, the compounds binding to the microtubules, and cell-cycle arrest [106].

Chicory plant has exhibited antifungal properties; nevertheless, the plant’s root methanol extract has been reported to have anti-cancer properties by the presence of various classes of compounds, such as lactucin, β-sitosterol, quinic acid, succinic acid, and polyphenols such as flavonoids in the root methanol extracts [70,72]. Flavonoids have been identified to have anti-cancer properties due to the compound’s ability to inhibit fatty acid synthase enzyme, which over-express and are highly active in cancer cells. From one study, it was found that the methanol root extract of chicory plant was able to reduce the viability of the human breast cancer SKBR3 cell line at concentrations of 400 µg/mL, 500 µg/mL, and 600 µg/mL, in a time- and concentration-dependent manner [70]. As the dose of the chicory methanol extract concentration and time duration of the exposure increases, the percentage of breast cancer cell viability decreases, thus indicating the applicability of chicory root methanol extract for anti-cancer treatment.

### 4.3. Anti-Inflammation and Anti-Diabetic Activity

Anti-inflammation is an important aspect which enhances the immune system of the host when the host is invaded by pathogens. However, inflammation may cause diseases such as heart disease, diabetic, cancer, and arthritis [114]. This issue can be overcome by consuming a diet which includes flavonoids and phenolic compounds that possess antioxidant activities [115]. Plant extracts isolated from the Middle East region have exhibited anti-inflammation and anti-diabetic properties, which aid in enhancing the immune system of the host.

Date fruits have been shown to exhibit an anti-inflammatory response due to the presence of phenolic compounds and flavonoids [116]. In a study, 29 different dates were tested for anti-inflammation activity. Water and methanol extracts were obtained for all the 29 varieties of the date fruits. 3-(4,5-dimethylthiazol-2-yl)-2,5-diphenyl tetrazolium bromide assay (MTT antioxidant assay) was conducted to identify compounds which were able to either reduce or remove the oxidative agents. From the study, it was found that all methanolic extracts at a concentration of 250 µg/mL showed better activity compared to the water extracts at a concentration of 250 µg/mL, where methanolic extracts of Khashram, Ruthana, and Luban showed the highest antioxidant activities. The anti-inflammatory effect was studied by the inhibition of cyclooxygenase enzymes—COX-1 and COX-2 enzymes—at 100 µg/mL for both water and methanolic extracts of all 29 varieties. Both the enzymes were inhibited by the water and methanolic extracts of all 29 varieties of dates, however the extracts showed higher inhibition of the COX-2 enzyme than the COX-1 enzyme, and methanolic extracts showcased better activity than the water extracts. The COX enzymes are responsible for production of prostaglandins, which promote inflammation [117]. From the study, it can be deduced that both water and methanol extracts from date fruits have the ability to inhibit COX enzymes, which are involved in the inflammation.

Methylprednisolone is a drug consumed for treatment of arthritis, blood disorders, allergic reactions, and eye conditions, to name a few. The consumption of this drug results in side effects which include affecting the neurotransmitter content in the brain [118]. In one study, the date seeds were obtained from Saudi Arabia and tested on male albino rats to determine the role of the seeds in reducing the side effect of methylprednisolone on the neurotransmitter content in the brain and also the level of testosterone in the rats [51]. The rats which were orally fed with date seeds of 20 mg/kg daily showed an increase in dopamine, gamma-aminobutyric acid, and norepinephrine (neurotransmitters) in the brain. The neurotransmitter dopamine is a chemical released by neurons to transmit an electrical signal between the neurons from the central nervous system, and gamma-aminobutyric acid acts as an inhibitor, to calm the activity of the nervous system, whereas norepinephrine is responsible for activity such as attentiveness, emotions, sleeping, dreaming, and learning [119,120,121]. An increase in these neurotransmitters indicated reduced chances for obtaining diseases such as Parkinson’s disease, Huntington’s disease, and mood disorders. Similarly, an increase in testosterone level was observed when the rats were fed with date fruit seeds.

Cactus has been identified to have anti-diabetic properties due to the presence of compounds such as linoleic acid (polyunsaturated fatty acid), oleic acid (monounsaturated fatty acid), vitamins such as tocopherols and vitamin K1, sterols, and carotenoids [122]. A study was conducted to study the effect of cactus pear seed oil on hypoglycemic and antihyperglycemic and also toxic effects of the oil on the diabetic and non-diabetic rats [122]. The rats were fed the seed oil orally and by intraperitoneal administration and there were no mortality, behavioral, or autonomic effects observed at dosages of 1 mL/kg, 3 mL/kg, or 5 mL/kg. The oral administration of the seed oil at 1 mL/kg or 2 mL/kg did not show any significant effect on the fasting blood glucose level of the healthy rats, but glibenclamide (drug to promote secretion of insulin) induced a drastic decrease in the blood glucose level of healthy rats. Other than that, in healthy rats, hyperglycemia was inhibited when the rats were orally administered with 0.8 mL/kg of the seed oil, whereas in streptozotocin-induced diabetic rats (STZ-diabetic rats) oral administration of the seed oil managed to improve the glucose tolerance in the diabetic-induced rats. Hence, this study showed that cactus pear seed oil has anti-diabetic properties by reducing the hyperglycemic effect in both normal and STZ-diabetic rats. The fatty acids found in the cactus pear seed oil were able to increase the secretion of the insulin, enhancing the fluidity of the cell membrane and GLUT4 transporter expression, thus enhancing the glucose-induced insulin secretion [122].

## 5. Conclusions

From this review, it has been shown that plants from the Middle Eastern region are able to exhibit various bioactivities, such as antimicrobial, anticancer, and antitumor activities; they are also rich in antioxidants. There are studies being conducted from the plants this region to obtain the analysis of phytochemicals from various parts of plants. These extracts from plants should be further studied to identify the composition and also their bioactivity as an individual compound. In future, isolated compounds from these plants can be tested along with current known antimicrobials to identify the ability of the compound to improve the efficacy of the currently-used drugs against antimicrobial resistant pathogens through synergistic interaction, and as well to determine the efficacy of chemotherapeutic drugs when combined with these isolated compounds against cancer and or tumor cells. All the compounds which have been identified to exhibit bioactivity can be isolated and clinical trials need to be conducted to determine the efficacy of these compounds as antimicrobial drugs. The recognition and trial of compounds which have anti-cancer or anti-tumor activities will aid in the reduction of the side effects caused by the current chemotherapy drugs. However, the challenge involved in Middle Eastern region plants would be the replication of extreme weather when the compounds are to be extracted. It is important to continue research in this region’s plants by utilizing traditional medicine as a reference to overcome the challenges in modern medicine, such as the lack of antimicrobial, antimalarial, and anti-cancer compounds.

## Figures and Tables

**Table 1:** Techniques to identify and separate the compounds found in the plants extracts [45,46,47].

**Chromatography**	Thin-layer chromatography
	Gas chromatography
	High-resolution liquid chromatography
	Capillary-liquid chromatography
**Electrophoresis**	Thin-layered electrophoresis
	Isotachophoresis
	Capillary electrophoresis
**Spectroscopic**	UV spectroscopy
	Infrared spectroscopy
	Near infrared spectroscopy
	Nuclear magnetic resonance spectroscopy
	Mass spectroscopy

**Table 2:** Compounds obtained from secondary metabolites of Middle East plants and their bioactivity.

Plants	Source of the Extract and The Compounds Extracted	Bioactivity	Extraction Method	Detection Method	Reference
*Phoenix dactylifera* (Date)	Seed of the date water extract: p-coumaric, ferulic, sinapic acids, and flavonoids procyanidins. Leaves of the date methanol extract: flavonoids. Pulp of Ajwa date seed extracted using ethanol: β-D-glucan, oleic-lauric fatty acid, linoleic acid, and palmitic acid.	Antibacterial activity against *Klebsiella pneumniae*, *Escherichia coli*, *Staphylococcus aureus*, *Staphylococcus epidermidis*, *Enterococcus faecalis*, and *Bacillus cereus*. Induce apoptosis on human hepatocellular carcinoma cells, boost immune systems.	The water extract of date seed was obtained using the solvent extraction method. The methanol leaf extract was obtained using the solvent extraction method. The ethanol leaf extract was obtained using the solvent extraction method.	Analysis was conducted using Agilent 1100 High-performance liquid chromatography (HPLC) System coupled with Bruker Esquire-LC ion trap mass spectrometer. The compounds were identified using thin layer chromatography by comparing with the standard color of gallic acid using an ultraviolet light lamp at 302 nm. The extract of Ajwa pulp was determined using High-performance liquid chromatography (HPLC) on a Waters 515 HPLC Pump System equipped with a Waters 2998 PDA Detector. For the chromatographic analysis, XBridge C_18_ Reverse Phase Column was utilized, where for mobile phase, two solvents, water and methanol-acetonitrile, were used. The HPLC was monitored at 254 nm to provide real-time chromatograms of the standard and the extract. β-D-glucan was used as the standard.	[48,49,50,51,52,53]
*Scrophularia umbrosa* (Green figwort)	Rhizome of the dichloromethane extract of plant: linoleic acid and palmitic acid. Seeds and leaves were extracted using water: iridoid glycosides. Hydro-alcoholic extract of the seed and aerial part of the plant: phenylethanoids, phenylpropanoids, flavonoids.	Antimalarial activity. Anti-cancer in Human Astrocytoma Cell Line (1321 and human breast cancer cell line. Antimicrobial activity against *Actinomyces viscous Streptococcus mutans*, *Streptococcus sobrinus*, *Lactobacillus fermentum*, *Lactobacillus casei* subsp *casei*, and *Eikenella corrodens*.	The extraction method conducted was Soxhlet apparatus. The extract was obtained using solvent extraction method. The extraction was performed using Soxhlet apparatus.	Dichloromethane extract was analyzed using Shimadzu GCMS-QP5050A gas chromatography-mass spectrometry (GC-MS) fitted with fused methyl silicon DB1 column where helium is used as carrier gas. The compounds were identified using gas chromatography silica gel H and C_18_ reversed phase silica gel along with thin layer chromatography which is pre-coated silica gel plates. The compounds were identified using column chromatography on silicagel 60 (Merck). The solvent systems used were *n*-hexane/ethyl acetate and ethyl acetate/methanol.	[54,55,56,57,58,59]
*Opuntia ficus-indica* (Cactus)	Root extract of the cactus using methanol: quercetin and silibin. Carotenes, α-cryptoxanthin, and β-carotene.	Anti-inflammatory and antioxidant activity. Protection against oxidative damage.	The extraction method applied was the solvent extraction method.	The compounds were identified using reverse phase chromatography and the compounds were separated using the retention time. The mobile phase consists of acetic acid and acetonitrile. The detection of the compound was set at 350 nm. The products were identified by ultraviolet-visible spectra and electrospray ionization mass spectra.	[60,61,62]
*Salvadora persica* (Meswak)	Stem region of the plant extracted using distilled water, ethanol, ethyl acetate and acetic acid: *N*-benzylbenzamide, decane, and stigmasterol. Roots and stems of the plant: Silica, tannins, and resins. Stem region of plant extracted using distilled water, ethyl acetate, ethanol, and acetic acid: *N-*benzylbenzamide, decane, and stigmasterol. Ethanol extract of the stick of the plant: Benzyl nitrate and benzylisothiocyanate. Stem of Meswak extracted using ethanol and the ethanol-free extract further extracted in chloroform: sterols, tannins, coumarins, alkaloids, and glycosides	Antibacterial activity against *Actinobacillus actinomycetemcomitans, Actinomyces naeslundii*, *Lactobacillus acidophilus*, *Porphyromonas gingivalis*, *Prevotella intermedia* and *Streptococcus mutans*. Oral hygiene by removing stains from teeth, protection against caries and gingivitis. Anti-fungal activity against *Candida albicans.* Antiviral agent against Herpes simplex virus type 1. Anti-tumor property against murine mouse melanoma where the growth of tumor was delayed.	The extraction method used was Soxhlet method. The extracts were obtained using the solvent extraction method. The extracts were obtained using the solvent extraction method. The extraction method employed was Soxhlet apparatus.	The compounds were identified using gas chromatography-mass spectrometry (GC-MS) analysis using a Hewlett-Packard HP 5890 series II chromatograph interfaced with a Fisons VG-7070E mass spectrometer. Helium gas was used as a carrier gas. The compounds were identified using gas chromatography-mass spectrometry (GC-MS) analysis interfaced with a Fisons VG-7070E mass spectrometer. The extract was fractionated using column chromatography, where *n*-hexane/ethyl acetate was used for elution and the compounds were identified using thin layer chromatography. Flash chromatography was performed, followed by recording of ultraviolet spectra on a spectrometer. The infrared spectra of the compounds were measured. Then, thin layer chromatography was performed using petroleum ether: chloroform: ethanol: acetic acid.	[63,64,65,66,67]
*Cichorium intybus* (Chicory)	Leaves: Lactucin, lactucopicrin, deoxylactucin, and β-1,3-dihydrolactucin. Roots: Lactucin, β-sitosterol, quinic acid, succinic acid, and polyphenols (flavonoids).	Antifungal activity against *Candida glabrata* and *Candida krusei*. Anti-cancer activity recorded against human breast cancer SKBR3 cell line.	The extraction was done using the solvent extraction method. The extraction was done using the maceration method.	The compounds were identified using Agilent 6550 iFunnel Q- Time-of-flight/mass spectrometer (Q-TOF/MS) system for chromatography analysis, followed by mass spectrometry conducted by Agilent 6550 Q-TOF/MS with an electrospray ionization source. The mobile phase at chromatography analysis utilized solvents formic acid in water and formic acid in acetonitrile. The extract was analyzed using High-performance liquid chromatography (HPLC) equipped with a photodiode array UV-vis detector SPD-M20A. Then, Liquid chromatography/mass spectrometry (LC/MS) analysis was conducted using a Quattro IItandem quadrupole mass spectrometer, fitted with an electrospray ionization source, followed byTandem mass spectrometry (MS/MS) analysis, which was done by transmitting precursor ions through mass spectrometry (MS) to the collision cell.	[68,69,70,71,72,73]
*Satureja thymbra* (Savory of crete)	Leaves soaked in ethanol: Thymol, carvacrol, *p*-cymene, γ-terpinene, borneol, caryophyllene, and bicyclogermacrene.	Anti-viral against Herpes simplex virus 1.	The extract was obtained using the solvent extraction method.	The analysis to determine the composition was carried out using gas chromatography system (Hewlett-Packard Co., model 6890), coupled with a selective mass detector (Hewlett Packard 5973), where electron impact ionization was carried out.	[74,75]

## References

[1] Hajar R. (2015). History of Medicine Timeline. Hear. Views.

[2] Isenberg H.D. (2003). Clinical Microbiology: Past, Present, and Future. J. Clin. Microbiol..

[3] Blevins S.M., Bronze M.S. (2010). Robert Koch and the “golden Age” of Bacteriology. Int. J. Infect. Dis..

[4] Beall A. (2018). Treating Cancer: Tackling Drug Resistance. Technol. Netw..

[5] Carter A. (2019). Chemotherapy: What It Is, What to Expect, Side Effects, and Outlook. Medical News Today. https://www.medicalnewstoday.com/articles/158401.php.

[6] Mahizan N.A., Yang S.K., Moo C.L., Song A.A.L., Chong C.M., Chong C.W., Abushelaibi A., Erin Lim S.H., Lai K.S. (2019). Terpene Derivatives as a Potential Agent against Antimicrobial Resistance (AMR) Pathogens. Molecules.

[7] Moo C.L., Yang S.K., Yusoff K., Ajat M., Thomas W., Abushelaibi A., Lim S.H.E., Lai K.S. (2019). Mechanisms of Antimicrobial Resistance (AMR) and Alternative Approaches to Overcome AMR. Curr. Drug Discov. Technol..

[8] Ritchie H., Roser M. Causes of Death. https://ourworldindata.org/causes-of-death.

[9] World Health Organization New Report Calls for Urgent Action to Avert Antimicrobial Resistance Crisis. https://www.who.int/news-room/detail/29-04-2019-new-report-calls-for-urgent-action-to-avert-antimicrobial-resistance-crisis.

[10] Walsh F. Superbugs to Kill “More Than Cancer” by 2050. https://www.bbc.com/news/health-30416844.

[11] Ringgaard A. (2014). What Are the Major Challenges to Modern Medicine? Science Nordic. https://sciencenordic.com/antibiotics-denmark-illness/what-are-the-major-challenges-to-modern-medicine/1404651.

[12] Yang S.K., Low L.Y., Yap P.S.X., Yusoff K., Mai C.-W., Lai K.S., Lim S.H.E. (2018). Plant-Derived Antimicrobials: Insights into Mitigation of Antimicrobial Resistance. Rec. Nat. Prod..

[13] Moo C.L., Yang S.K., Osman M.A.J.Y.L., Lim W.M., Lim S.H.E., Lai K.S. (2019). Antibacterial Activity and Mode of Action of β -Caryophyllene on Bacillus Cereus. Pol. J. Microbiol..

[14] Yang S.K., Yusoff K., Warren T., Akseer R., Alhosani M.S., Abushelaibi A., Lim S.H.E., Lai K.S. (2019). Lavender Essential Oil Induces Oxidative Stress Which Modifies the Bacterial Membrane Permeability of Carbapenemase Producing Klebsiella Pneumoniae. Sci. Rep..

[15] Yang S.K., Yusoff K., Ajat M., Thomas W., Abushelaibi A., Akseer R., Lim S.H.E., Lai K.S. (2019). Disruption of KPC-Producing *Klebsiella pneumoniae* Membrane via Induction of Oxidative Stress by Cinnamon Bark (*Cinnamomum Verum* J. Presl) Essential Oil. PLoS ONE.

[16] Yap P.S.X., Yiap B.C., Ping H.C., Lim S.H.E. (2014). Essential Oils, A New Horizon in Combating Bacterial Antibiotic Resistance. Open Microbiol. J..

[17] Yang L., Wen K.S., Ruan X., Zhao Y.X., Wei F., Wang Q. (2018). Response of Plant Secondary Metabolites to Environmental Factors. Molecules.

[18] Isah T. (2019). Stress and Defense Responses in Plant Secondary Metabolites Production. Biol. Res..

[19] Pariano A. Which Are the Middle Eastern Countries? WorldAtlas.com. https://www.worldatlas.com/articles/which-are-the-middle-eastern-countries.html.

[20] Global Banking and Finance. List of Countries in the Middle East—Global Banking; Finance Review. https://www.globalbankingandfinance.com/list-of-countries-in-the-middle-east/.

[21] Ghazanfar S.A., McDaniel T. (2016). Floras of the Middle East: A Quantitative Analysis and Biogeography of the Flora of Iraq. Edinburgh J. Bot..

[22] Sani N.M., Abdulkadir F., Mujahid N.S. (2018). Antimicrobial Activity of *Phoenix dactylifera* (Date Palm) on Some Selected Members of Enterobacteriaceae. Bayero J. Pure Appl. Sci..

[23] Yuan H., Ma Q., Ye L., Piao G. (2016). The Traditional Medicine and Modern Medicine from Natural Products. Molecules.

[24] Fabricant D.S., Farnsworth N.R. (2001). The Vaue of Plants Used in Traditional Medicine for Drug Discovery. Environ. Health Perspect..

[25] World Health Organization (2000). Traditional and Modern Medicine.

[26] Alves R.R.N., Rosa I.M.L. (2007). Biodiversity, Traditional Medicine and Public Health: Where Do They Meet?. J. Ethnobiol. Ethnomed..

[27] Ekor M. (2014). The Growing Use of Herbal Medicines: Issues Relating to Adverse Reactions and Challenges in Monitoring Safety. Front. Pharmacol..

[28] Foster D.F., Phillips R.S., Hamel M.B., Eisenberg D.M. (2000). Alternative Medicine Use in Older Americans. J. Am. Geriatr. Soc..

[29] Bodecker G., Ong C.-K., Grundy C., Burford G., Shein K. (2005). WHO Global Atlas of Traditional, Complementary and Alternative Medicine.

[30] Bandaranayake W.M., Ahmad I., Aqil F., Owais M. (2006). Quality Control, Screening, Toxicity, and Regulation of Herbal Drugs. Modern Phytomedicine: Turning Medicinal Plants into Drugs.

[31] Mukherjee Pulok K., Shiv B., Harwansh Ranjit K., Chaudhary Sushil K. (2014). Shifting Paradigm for Validation of Medicinal Plants in Indian Traditional Medicine. Indian Drugs.

[32] Bimonte S., Barbieri A., Palma G., Arra C. (2013). The Role of Morphine in Animal Models of Human Cancer: Does Morphine Promote or Inhibit the Tumor Growth?. Biomed. Res. Int..

[33] Joo Y.E. (2014). Natural Product-Derived Drugs for the Treatment of Inflammatory Bowel Diseases. Intest. Res..

[34] Dwarakanath C. Use of Opium and Cannabis in the Traditional Systems of Medicine in India. https://www.unodc.org/unodc/en/data-and-analysis/bulletin/bulletin_1965-01-01_1_page004.html.

[35] Krishnamurti C., Rao S.S.C.C. (2016). The Isolation of Morphine by Serturner. Indian J. Anaesth..

[36] Corson T.W., Crews C.M. (2007). Molecular Understanding and Modern Application of Traditional Medicines: Triumphs and Trials. Cell.

[37] Atanasov A.G., Waltenberger B., Pferschy-Wenzig E.M., Linder T., Wawrosch C., Uhrin P., Temml V., Wang L., Schwaiger S., Heiss E.H. (2015). Discovery and Resupply of Pharmacologically Active Plant-Derived Natural Products: A Review. Biotechnol. Adv..

[38] Crowden C.J., Paterson I. (1997). Cancer Drugs Better than Taxol?. Nature.

[39] Varijakzhan D., Yang S.K., Chong C.M., Akseer R., Alhosani M.S., Thomas W., Lai K.S., Lim S.H.E. (2020). Essential Oils as Potential Antimicrobial Agents.

[40] Rassem H.H.A., Nour A.H., Yunus R.M. (2016). Techniques for Extraction of Essential Oils from Plants: A Review. Aust. J. Basic Appl. Sci..

[41] Aziz Z.A.A., Ahmad A., Setapar S.H.M., Karakucuk A., Azim M.M., Lokhat D., Rafatullah M., Ganash M., Kamal M.A., Ashraf G.M. (2018). Essential Oils: Extraction Techniques, Pharmaceutical and Therapeutic Potential—A Review. Curr. Drug Metab..

[42] Hassim N., Markom M., Anuar N., Baharum S.N. (2014). Solvent Selectio in Extraction of Essential Oil and Bioactive Compounds from *Polygonum minus*. Appl. Sci..

[43] Tongnuanchan P., Benjakul S. (2014). Essential Oils: Extraction, Bioactivities, and Their Uses for Food Preservation. J. Food Sci..

[44] Mendoza N., Silva E.M.E., Asao T., Asaduzzaman M. (2018). Introduction to Phytochemicals: Secondary Metabolites from Plants with Active Principles for Pharmacological Importance. Phytochemicals: Source of Antioxidants and Role in Disease Prevention.

[45] Lahlou M. (2004). Methods to Study the Phytochemistry and Bioactivity of Essential Oils. Phyther. Res..

[46] Yap P.S.X., Krishnan T., Chan K.G., Lim S.H.E. (2015). Antibacterial Mode of Action of *Cinnamomum verum* Bark Essential Oil, Alone and in Combination with Piperacillin, against a Multi-Drug-Resistant *Escherichia coli* Strain. J. Microbiol. Biotechnol..

[47] De Souza A.V.V., Dos Santos U.S., de Sá Carvalho J.R., Barbosa B.D.R., Canuto K.M., Rodrigues T.H.S. (2018). Chemical Composition of Essential Oil of Leaves from *Lippia Schaueriana* Mart. Collected in the Caatinga Area. Molecules.

[48] Yasin B.R., El-Fawal H.A.N., Mousa S.A. (2015). Date (*Phoenix dactylifera*) Polyphenolics and Other Bioactive Compounds: A Traditional Islamic Remedy’s Potential in Prevention of Cell Damage, Cancer Therapeutics and Beyond. Int. J. Mol. Sci..

[49] Bentrad N., Gaceb-Terrak R., Benmalek Y., Rahmania F. (2017). Studies on Chemical Composition and Antimicrobial Activities of Bioactive Molecules from Date Palm (*Phoenix dactylifera* L.) Pollens and Seeds. African J. Tradit. Complement. Altern. Med. AJTCAM.

[50] Al-Farsi M., Alasalvar C., Al-Abid M., Al-Shoaily K., Al-Amry M., Al-Rawahy F. (2007). Compositional and Functional Characteristics of Dates, Syrups, and Their by-Products. Food Chem..

[51] Saddiq A.A., Bawazir A.E. (2010). Antimicrobial Activity of Date Palm (*Phoenix dactylifera*) Pits Extracts and Its Role in Reducing the Side Effect of Methyl Prednisolone on Some Neurotransmitter Content in the Brain, Hormone Testosterone in Adulthood. Acta Hortic..

[52] Gu L., Kelm M.A., Hammerstone J.F., Beecher G., Holden J., Haytowitz D., Prior R.L. (2003). Screening of Foods Containing Proanthocyanidins and Their Structural Characterization Using LC-MS/MS and Thiolytic Degradation. J. Agric. Food Chem..

[53] Siddiqui S., Ahmad R., Khan M.A., Upadhyay S., Husain I., Srivastava A.N. (2019). Cytostatic and Anti-Tumor Potential of Ajwa Date Pulp against Human Hepatocellular Carcinoma HepG2 Cells. Sci. Rep..

[54] Nikkhah E., Afshar F.H., Babaei H., Delazar A., Asgharian P. (2018). Evaluation of Phytochemistry and Some Biological Activities of Aerial Parts and Seed of *Scrophularia umbrosa* Dumort. Jundishapur J. Nat. Pharm. Prod..

[55] Nikkhah E., Afshar F.H., Babaei H., Asgharian P., Delazar A. (2018). Phytochemical Analysis and In-Vitro Bioactivity of *Scrophularia umbrosa* Rhizome (Scrophulariaceae). Iran. J. Pharm. Res..

[56] Fernández M.A., García M.D., Sáenz M.T. (1996). Antibacterial Activity of the Phenolic Acids Fractions of *Scrophularia frutescens* and *Scrophularia sambucifolia*. J. Ethnopharmacol..

[57] Lajimi A.A., Rezaie-Tavirani M., Mortazavi S.A., Barzegar M., Moghadamnia S.H., Rezaee M.B. (2010). Study of Anti Cancer Property of *Scrophularia striata* Extract on the Human Astrocytoma Cell Line (1321). Iran. J. Pharm. Res..

[58] Pasdaran A., Hamedi A. (2017). The Genus Scrophularia: A Source of Iridoids and Terpenoids with a Diverse Biological Activity. Pharm. Biol..

[59] Chen B., Liu Y., Lu H.W., Wang N.L., Yang B.F., Yao X.S. (2008). Iridoid and Aromatic Glycosides from *Scrophularia ningpoensis* Hemsl. and Their Inhibition of [Ca^2+^]_i_, Increase Induced by KCI. Chem. Biodivers..

[60] Slimen B. (2016). *Opuntia ficus-indica* as a Source of Bioactive and Nutritional Phytochemicals. J. Food Nutr. Sci..

[61] Alimi H., Hfaiedh N., Bouoni Z., Hfaiedh M., Sakly M., Zourgui L., Rhouma K. (2010). Ben. Antioxidant and Antiulcerogenic Activities of *Opuntia ficus indica* f. Inermis Root Extract in Rats. Phytomedicine.

[62] Weirong C.A.I., Xiaohong G.U., Tang J. (2010). Extraction, Purification, and Characterisation of the Flavonoids from *Opuntia milpa* Alta Skin. Czech J. Food Sci..

[63] AbdELRahman H.F., Skaug N., Whyatt A.M., Francis G.W. (2003). Volatile Compounds in Crude *Salvadora persica* Extracts. Pharm. Biol..

[64] EL-Hefny M., Ali H.M., Ashmawy N.A., Salem M.Z.M. (2017). Chemical Composition and Bioactivity of *Salvadora persica* Extracts against Some Potato Bacterial Pathogens. BioResources.

[65] Ahmad H., Rajagopal K. (2012). Biological Activities of *Salvadora persica* L. (Meswak). Med. Aromat. Plants.

[66] Khalil A.T. (2006). Benzylamides from *Salvadora persica*. Arch. Pharm. Res..

[67] Iyer D., Patil U.K. (2014). Evaluation of Antihyperlipidemic and Antitumor Activities of Isolated Coumarins from *Salvadora indica*. Pharm. Biol..

[68] Badakhasann S., Bhatnagar S. (2019). Cichorium Intybus an Anti-Fungal Drug: A Prospective Study in Tertiary Care Hospital of Kashmir Valley. ACTA Sci. Microbiol..

[69] Eslami H., Babaei H., Falsafi P., Rahbar M., Najar-Karimi F., Pourzare-Mehrbani S. (2017). Evaluation of the Antifungal Effect of Chicory Extracts on *Candida glabrata* and *Candida krusei* in a Laboratory Environment. J. Contemp. Dent. Pract..

[70] Mehrandish R., Awsat Mellati A., Rahimipour A., Dehghan Nayeri N. (2017). Anti-Cancer Activity of Methanol Extracts of *Cichorium intybus* on Human Breast Cancer SKBR3 Cell Line. Razavi Int. J. Med..

[71] Bian M., Lin Z., Wang Y., Zhang B., Li G., Wang H. (2018). Bioinformatic and Metabolomic Analysis Reveal Intervention Effects of Chicory in a Quail Model of Hyperuricemia. Evid. Based Complement. Altern. Med..

[72] Li B.H., Tian W.X. (2004). Inhibitory Effects of Flavonoids on Animal Fatty Acid Synthase. J. Biochem..

[73] Bahri M., Hance P., Grec S., Quillet M.C., Trotin F., Hilbert J.L., Hendriks T. (2012). A “Novel” Protocol for the Analysis of Hydroxycinnamic Acids in Leaf Tissue of Chicory (*Cichorium intybus* L., Asteraceae). Sci. World J..

[74] Tepe B., Cilkiz M. (2016). A Pharmacological and Phytochemical Overview on *Satureja*. Pharm. Biol..

[75] Loizzo M.R., Saab A.M., Tundis R., Statti G.A., Menichimi F., Lampronti D., Gambari R., Cinatl J., Doerr H.W. (2008). Phytochemical Analysis and in Vitro Antiviral Activities of the Essential Oils of Seven Lebanon Species. Chem. Biodivers..

[76] Mazid M., Khan T.A., Mohammad F. (2011). Role of Secondary Metabolites in Defense Mechanisms of Plants. Biol. Med..

[77] Chassagne F., Cabanac G., Hubert G., David B., Marti G. (2019). The Landscape of Natural Product Diversity and Their Pharmacological Relevance from a Focus on the Dictionary of Natural Products^®^. Phytochem. Rev..

[78] Do Q.D., Angkawijaya A.E., Tran-Nguyen P.L., Huynh L.H., Soetaredjo F.E., Ismadji S., Ju Y.H. (2014). Effect of Extraction Solvent on Total Phenol Content, Total Flavonoid Content, and Antioxidant Activity of *Limnophila aromatica*. J. Food Drug Anal..

[79] Bouarab-Chibane L., Forquet V., Lantéri P., Clément Y., Léonard-Akkari L., Oulahal N., Degraeve P., Bordes C. (2019). Antibacterial Properties of Polyphenols: Characterization and QSAR (Quantitative Structure-ativity Relationship) Models. Front. Microbiol..

[80] Khan G.A. (2018). Dates: A Middle Eastern Delicacy. Arab News. https://www.arabnews.com/node/1301551/saudi-arabia.

[81] Qadoos H.A., Dhafari H.S., Al Marzooqi D.A., Kumarappan A., Nazir A. (2017). Phenolic Content and Antimicrobial Activities of Date Palm (*Phoenix dactylifera* L.) Fruits and Leaves. Food Biol..

[82] ALrajhi M., AL-Rasheedi M., Eltom S.E.M., Alhazmi Y., Mustafa M.M., Ali Al. M. (2019). Antibacterial Activity of Date Palm Cake Extracts (*Phoenix dactylifera*). Cogent Food Agric..

[83] Abdallah E., Musa K., Qureshi K., Sadeek A. (2017). Antimicrobial Activity and Antioxidant Potential of the Methanolic Leaf Extracts of Three Cultivars of Date Palm Trees (*Phoenix dactylifera*) from Saudi Arabia. Med. Sci. Int. Med. J..

[84] Islam M.Z., Hossain M.T., Hossen F., Mukharjee S.K., Sultana N., Paul S.C. (2018). Evaluation of Antioxidant and Antibacterial Activities of *Crotalaria pallida* Stem Extract. Clin. Phytoscience.

[85] Vahabi S., Najafi E., Alizadeh S. (2011). In Vitro Antimicrobial Effects of Some Herbal Essences against Oral Pathogens. J. Med. Plant Res..

[86] Dai J., Mumper R.J. (2010). Plant Phenolics: Extraction, Analysis and Their Antioxidant and Anticancer Properties. Molecules.

[87] Fu G., Pang H., Wong Y. (2008). Naturally Occurring Phenylethanoid Glycosides: Potential Leads for New Therapeutics. Curr. Med. Chem..

[88] Deng Y., Lu S. (2017). Biosynthesis and Regulation of Phenylpropanoids in Plants. CRC. Crit. Rev. Plant Sci..

[89] Doan L.P., Nguyen T.T., Pham M.Q., Tran Q.T., Pham Q.L., Tran D.Q., Than V.T., Bach L.G. (2019). Extraction Process, Identification of Fatty Acids, Tocopherols, Sterols and Phenolic Constituents, and Antioxidant Evaluation of Seed Oils from Five Fabaceae Species. Processes.

[90] Das A.B., Goud V.V., Das C. (2019). Phenolic Compounds as Functional Ingredients in Beverages. Value-Added Ingredients and Enrichments of Beverages.

[91] Zheng C.J., Yoo J.S., Lee T.G., Cho H.Y., Kim Y.H., Kim W.G. (2005). Fatty Acid Synthesis Is a Target for Antibacterial Activity of Unsaturated Fatty Acids. FEBS Lett..

[92] Dahiya P., Kamal R., Luthra R., Mishra R., Saini G. (2012). Miswak: A Periodontist′s Perspective. J. Ayurveda Integr. Med..

[93] Ahmad H., Rajagopal K. (2014). *Salvadora persica* L. (Meswak) in Dental Hygiene. Saudi J. Dent. Res..

[94] Abubacker M.N., Kokila K., Sumathi R. (2012). In Vitro Antimicrobial Effects of Crude Plant Chewing Sticks Extracts on Oral Pathogen. Biosci. Biotechnol. Res. Asia.

[95] Silva Junior I.F., Raimondi M., Zacchino S., Cechinel Filho V., Noldin V.F., Rao V.S., Lima J.C.S., Martins D.T.O. (2010). Evaluation of the Antifungal Activity and Mode of Action of *Lafoensia pacari* A. St.-Hil., Lythraceae, Stem-Bark Extracts, Fractions and Ellagic Acid. Rev. Bras. Farmacogn..

[96] Visintini Jaime M.F., Redko F., Muschietti L.V., Campos R.H., Martino V.S., Cavallaro L.V. (2013). In Vitro Antiviral Activity of Plant Extracts from Asteraceae Medicinal Plants. Virol. J..

[97] Taha M.Y. (2008). Antiviral Effect of Ethanolic Extract of *Salvadora Persica* (Siwak) on Herpes Simplex Virus Infection. Al–Rafidain Dent. J..

[98] Saab A.M., Lampronti I., Finotti A., Borgatti M., Gambari R., Esseily F., Safi S., Diab-Assaf M., Rabenau H., Cinatl J. (2012). In Vitro Evaluation of the Biological Activity of Lebanese Medicinal Plants Extracts against Herpes Simplex Virus Type 1. Minerva Biotecnol..

[99] Medini F., Megdiche W., Mshvildadze V., Pichette A., Legault J., St-Gelais A., Ksouri R. (2016). Antiviral-Guided Fractionation and Isolation of Phenolic Compounds from *Limonium densiflorum* Hydroalcoholic Extract. Comptes Rendus Chim..

[100] Astani A., Schnitzler P. (2014). Antiviral Activity of Monoterpenes Beta-Pinene and Limonene against Herpes Simplex Virus in Vitro. Iran. J. Microbiol..

[101] Suurbaar J., Mosobil R., Donkor A.M. (2017). Antibacterial and Antifungal Activities and Phytochemical Profile of Leaf Extract from Different Extractants of *Ricinus communis* against Selected Pathogens. BMC Res. Notes.

[102] Ambikapathy V., Gomathi S., Panneerselvam A. (2011). Effect of Antifungal Activity of Some Medicinal Plants against *Pythium debaryanum* (Hesse). Pelagia Res. Libr. Asian J. Plant Sci. Res..

[103] Mayer-Chissick U., Lev E., Yaniv Z., Dudai N. (2014). Wild Edible Plants in Israel Tradition Versis Cultivation. Medicinal and Aromatic Plants of the Middle-East.

[104] Shaikh T., Mujum A., Wasimuzzama K., Rub R. (2010). An Overview on Phytochemical and Pharmacological Profile of *Cichorium intybus* Linn. Pharmacol. Online.

[105] Kubo I. (1995). Antifungal Sesquiterpene Dialdehydes from the Warburgia Plants and Their Synergists. Stud. Nat. Prod. Chem..

[106] Lichota A., Gwozdzinski K. (2018). Anticancer Activity of Natural Compounds from Plant and Marine Environment. Int. J. Mol. Sci..

[107] Weinberg F., Ramnath N., Nagrath D. (2019). Reactive Oxygen Species in the Tumor. Cancers.

[108] Hussain H., Nazir M., Green I.R., Saleem M., Raza M.L. (2019). Therapeutic Potential of Iridoid Derivatives: Patent Review. Inventions.

[109] Orangi M., Pasdaran A., Shanehbandi D., Kazemi T., Yousefi B., Hosseini B.A., Baradaran B. (2016). Cytotoxic and Apoptotic Activities of Methanolic Subfractions of Scrophularia Oxysepala against Human Breast Cancer Cell Line. Evid. Based Complement. Altern. Med..

[110] Loizzo M.R., Bruno M., Balzano M., Giardinieri A., Pacetti D., Frega N.G., Sicari V., Leporini M., Tundis R. (2019). Comparative Chemical Composition and Bioactivity of *Opuntia ficus-indica sanguigna* and Surfarina Seed Oils Obtained by Traditional and Ultrasound-Assisted Extraction Procedures. Eur. J. Lipid Sci. Technol..

[111] Basli A., Belkacem N., Amrani I. (2017). Health Benefits of Phenolic Compounds against Cancers. Phenolic Compounds-Biological Activity.

[112] Berger A., Jones P.J.H., Abumweis S.S. (2004). Plant Sterols: Factors Affecting Their Efficacy and Safety as Functional Food Ingredients. Biomed Cent..

[113] Yildirim I., Kutlu T. (2015). Anticancer Agents: Saponin and Tannin. Int. J. Biol. Chem..

[114] Harvard’s Women Health Watch (2018). Foods That Fight Inflammation—Harvard Health.

[115] Zhen J., Guo Y., Villani T., Carr S., Brendler T., Mumbengegwi D.R., Kong A.N.T., Simon J.E., Wu Q. (2015). Phytochemical Analysis and Anti-Inflammatory Activity of the Extracts of the African Medicinal Plant *Ximenia caffra*. J. Anal. Methods Chem..

[116] Zhang C.R., Aldosari S.A., Vidyasagar P.S.P.V., Shukla P., Nair M.G. (2017). Health-Benefits of Date Fruits Produced in Saudi Arabia Based on In Vitro Antioxidant, Anti-Inflammatory and Human Tumor Cell Proliferation Inhibitory Assays. J. Saudi Soc. Agric. Sci..

[117] Fitzpatrick F. (2005). Cyclooxygenase Enzymes: Regulation and Function. Curr. Pharm. Des..

[118] Phan K., Xiong C., Daubs M.D., Tian H., Montgomery S.R., Aghdasi B., Suzuki A., Li J., Scott T., Wang J.C. (2013). The Anti-Inflammatory Effects of Perioperative Methylprednisolone on the Soft Tissue Inflammation Induced by RhBMP-2. Spine J..

[119] Fisch R.Z., Bannett J., Belmaker R.H. (2004). The Role of Norepinephrine in Schizophrenia. J. Neuropsychiatry Clin. Neurosci..

[120] Shelp B.J., Bown A.W., Mclean M.D. (1999). Metabolism and Functions of Gamma-Aminobutyric Acid. Trends Plant Sci..

[121] Juárez Olguín H., Calderón Guzmán D., Hernández García E., Barragán Mejía G. (2016). The Role of Dopamine and Its Dysfunction as a Consequence of Oxidative Stress. Oxid. Med. Cell. Longev..

[122] Berraaouan A., Ziyyat A., Mekhfi H., Legssyer A., Sindic M., Aziz M., Bnouham M. (2014). Evaluation of Antidiabetic Properties of Cactus Pear Seed Oil in Rats. Pharm. Biol..

